# Effect of sulodexide in patients with non-proliferative diabetic retinopathy: diabetic retinopathy sulodexide study (DRESS)

**DOI:** 10.1007/s00417-014-2746-8

**Published:** 2014-08-12

**Authors:** Ji Hun Song, Hee Seung Chin, Oh Woong Kwon, Su Jin Lim, Ha Kyoung Kim, for the DRESS Research Group

**Affiliations:** 1 Department of Ophthalmology, Ajou University School of Medicine, Suwon, Korea; 2 Department of Ophthalmology, Inha University School of Medicine, Incheon, Korea; 3 The Retina Center, Nune Eye Hospital, Seoul, Korea; 4 Department of Ophthalmology, Kangnam Sacred Heart Hospital, College of Medicine, Hallym University, #948-1 Daerim 1-dong, Yeongdeungpo-gu, Seoul, 150-950 Korea

**Keywords:** Diabetic retinopathy, Hard exudates, Randomized controlled trial, Sulodexide

## Abstract

**Purpose:**

To evaluate the effectiveness of sulodexide for the treatment of hard exudates (HE) in non-proliferative diabetic retinopathy (NPDR).

**Methods:**

This was a randomized, placebo-controlled, multicenter trial involving 130 patients (65 for each group) who had mild-to-moderate NPDR with macular HE. Participants were given a daily dose of either 50 mg sulodexide or a matching dose of placebo orally for 12 months. Main outcome measure was an improvement in HE defined as a decrease in severity by at least two grades on a 10-grade severity scale. This was evaluated by fundus photography over 12-month period.

**Results:**

The sulodexide group showed significantly greater improvement in HE severity than that shown by the placebo group (39.0 % vs. 19.3 %; chi square, *P* = 0.005). Logistic regression analysis yielded an odds ratio of 2.790 (95 % confidence interval, 1.155-6.743; *P* = 0.023) for the effect of treatment once adjustments were made for demographic, prognostic and disease confounders. Intention to treat and per-protocol analysis yielded similar results. Sulodexide’s safety was comparable to that of the placebo.

**Conclusions:**

Oral sulodexide therapy over 12 months improved macular HE in patients with mild-to-moderate NPDR, without leading to detectable adverse events. The study protocol was registered on clinicaltrial.gov under identifier NCT01295775.

## Introduction

Diabetic retinopathy is the leading cause of new onset blindness among the working-age group in industrialized countries, affecting from 2 to 5 % of the entire population [1]. The first microscopic changes are thickening of the retinal capillary basement membrane and degeneration of pericytes, both of which compromise the integrity of the capillary wall, followed by pericyte loss. Collagen progressively replaces the glycosaminoglycans (GAGs) of the basal membrane, leading to modifications in vascular permeability due to altered anionic charge. These changes eventually result in the clinical appearance of vascular leakage from the retinal capillaries followed by microaneurysms. If vascular leakage persists, serum proteins and lipids deposit in the retina and form hard exudates (HE).

The substitution of GAGs by collagen leads to a basal membrane thickening also in the kidney. As in the retina, these changes in permeability of the renal glomeruli induce the selective loss of proteins, clinically detected as albuminuria [2]. It has been suggested that altered GAG metabolism in diabetes patients could be a common pathogenic factor in diabetic vascular disease [3]. Evidence that both diabetic retinopathy and nephropathy have a common underlying pathogenesis (the depletion of GAGs from the basement membrane) is supported by the findings of the Wisconsin Epidemiologic Study of Diabetic Retinopathy in which micro-albuminuria was reported to be cross-sectionally associated with retinopathy in diabetes patients [4]. Since current anti-hyperglycemic therapy cannot fully prevent the complications associated with diabetes, it is imperative to find new drugs that can slow or reverse the vascular micro-abnormalities that ensue.

Sulodexide (Vessel Due F®, Aju Pharm, Seoul, South Korea, under license from Alfa Wassermann, Bologna, Italy) is a GAG that consists of heparin and dermatan sulfate, and is available for oral administration [5]. It has a high trophism for the vessel wall [6, 7]. Clinical studies have already shown its efficacy in decreasing micro- and macro-albuminuria in diabetes patients, thus suggesting that the partial restoration of glomerular GAG content might slow the progression of nephropathy [8–13]. Moreover, preliminary observations on the use of sulodexide in patients with diabetic retinopathy was a significant reduction in HE, thus indicating a potential benefit of this GAG at the retinal capillary level [14, 15].

On the basis of these pathophysiological considerations and experimental findings, we decided to evaluate the effects of sulodexide on diabetic retinopathy in a double-masked, randomized, placebo-controlled, multicenter trial: the Diabetic Retinopathy Sulodexide Study (DRESS). In this phase-2 study, we aimed to assess the effectiveness of sulodexide for the treatment of HE in non-proliferative diabetic retinopathy (NPDR), in patients with type 1 and type 2 diabetes mellitus (DM).

## Methods

### Study design

This was a double-masked, randomized, controlled, multicenter study consisting of 130 patients (65 for each treatment arm) with mild-to–moderate NPDR. Table 1 shows the selection criteria for the study population. At baseline (T0), ocular lesions were graded using color fundus photography and fluorescein angiography (FA). The photographs and FA images were subsequently sent to a review committee comprising off-site assessors (JH Song, SJ Lim, and HS Chin), who were unaware of the initial investigators’ assessment. This committee was nominated to confirm the quality of the images and the grade of the lesions. After this validation process, eligible patients were blindly allocated into the following two groups using a computer-generated list: test (sulodexide) and control (placebo) groups. Two daily oral dose of sulodexide 25 mg or the matching placebo were administered to each patient, one capsule in the morning and one capsule in the evening, for 360 ± 7 days. The selected dosage regimen for this study was based on previously published clinical trial data on sulodexide [7, 9, 11, 12, 16]. Patients were then evaluated at 3 (T3), 6 (T6), 9 (T9), and 12 months (T12) with a 1-week window allowed around the follow-up times. This study was approved by the institutional review board (IRB) and/or the ethics committee of each participating center, and conducted in accordance with the Declaration of Helsinki and the guidelines on good clinical practice. All participants signed the written informed consent in the form approved by the relevant IRB. The study protocol was registered on clinicaltrial.gov under identifier NCT01295775.

**Table 1:** Selection of study population

Inclusion criteria	Exclusion criteria
Patients aged over 18 years with type 1 or type 2 DM Diabetes under good control with drugs for at least 6 months (HbA1c <9 % [75 mmol/mol]) CFT ≤ 300 um of retina by OCT Snellen visual acuity ≥ 0.4 (20/50) Mild-to-moderate NPDR assessed by fundus photography and FA according to Airlie House Classification and ETDRS: (i) the presence of hard exudates within grade 2 and 5 (ii) at least one of the following lesions: vascular leakages, microaneuryms, hemorrhages, IRMA DBP ≤ 90 mmHg and SBP ≤ 130 mmHg with or without medication Controlled arterial blood pressure for the last 6 months	NPDR which is being treated with laser therapy or should be treated with laser therapy before the end of the study Laser therapy or intravitreal injection (anti-VEGF, steroid) within 3 months from enrollment Concomitant retinal disease due to causes other than diabetic microangiopathy Concomitant therapy (i) antihypertensive treatment, unless administered at stable dosage for at least 6 months before the start of the study (ii) ACE inhibitor/ARB therapy, unless administered at stable dosage for at least 6 months before the start of the study (iii) warfarin therapy (iv) hemorrheological, vasoactive drugs and antithrombotics except acetylsalicylic acid at stable dosage Severe liver impairment (CHILD C) Severe renal insufficiency (creatinine >2.2 mg/dl) Severe cardiac insufficiency (NYHA - New York Heart Association classes 3 – 4) Clinical history of diathesis and haemorrhagic disease Individual hypersensitivity toward the product, heparin, low molecular weight heparin, or heparin-like products Intended or ascertained pregnancy or lactation Participation in a trial within the past 6 months Surgery or trauma within the past 6 months Planned surgical intervention within 6 months from enrolment

ACE = angiotensin-converting enzyime, ARB = angiotensin-receptor blocker, CFT = central foveal thickness, DBP = diastolic blood pressure, DM = diabetes mellitus, ETDRS = early treatment diabetic retinopathy study, FA = fluorescein angiography, IRMA = intraretinal microvascular abnormalities, NPDR = non-proliferative diabetic retinopathy, OCT = optical coherence tomography, SBP = systolic blood pressure, VEGF = vascular endothelial growth factor

### Ophthalmic evaluation

A comprehensive ophthalmic examination was performed during every visit, including assessment of best-corrected visual acuity (BCVA) using the Early Treatment Diabetic Retinopathy Study (ETDRS) protocol; intraocular pressure (IOP) measurement; slit lamp biomicroscopy; and indirect ophthalmoscopy. Fundus photography and a contrast sensitivity test (CST) using the Mars Letter CST (Mars Perceptrix, Chappaqua, NY, USA), were performed during every visit. Optical coherence tomography (OCT) and FA were performed at T0, T6, and T12. Macular thickness was measured by OCT (Stratus OCT, Carl Zeiss Meditec, Dublin, CA, USA), and changes in the mean 1-mm central foveal thickness (CFT) served to evaluate changes in macular edema.

### Efficacy end-points

**Fig. 1:**
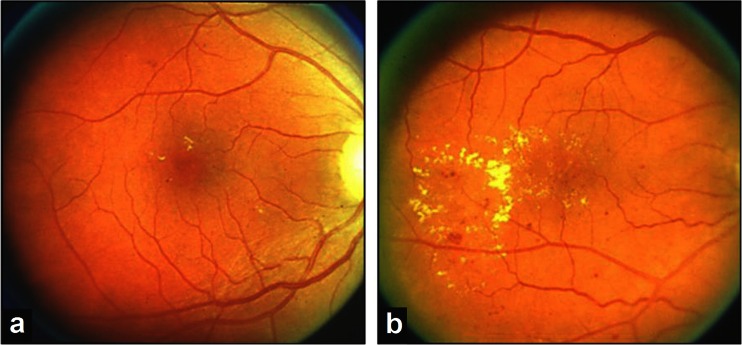
The early treatment diabetic retinopathy study (ETDRS) standard photographs 3 (**a**) and 4 (**b**). From the Early Treatment Diabetic Retinopathy Study Research Group (1991); Grading diabetic retinopathy from stereoscopic color fundus photographs – an extension of the modified Airlie House classification. Ophthalmology 98:786–806. Reprinted courtesy of Elsevier

The primary efficacy endpoint of this study was an improvement of HE, defined as a decrease in HE by at least two categories of severity. Fundus photography was performed on fields F1 and F2 (ETDRS Standard), and the HE were graded according to the specific grading system of this study which uses an extension of the Airlie House classification, while FA was performed on seven standard fields (ETDRS Standard) and graded according to ETDRS [17, 18]. The grading of HE was based on the area of retina involved and the amount of HE observed, using ETDRS standard photographs 3 and 4 for comparison, as follows (Fig. 1);

#### Grading of hard exudates

1. Questionable HE
2. Definite HE; fewer than those in standard photograph 3
3. HE; as many as those in standard photograph 3
4. HE; more than those in standard photograph 3, but fewer than those in standard photograph 4
5. HE; more than or as many as those in standard photograph 4

Since the grade 4 fundus photographs included too wide a range, it was divided into grade 4a and 4b, which in turn were divided into “−”, “0”, and “+” grades in order to measure adequately the changes in HE.

Grade 4a- Very mild deposition of scattered HE but more than those in standard photograph 3
Grade 4a Mild deposition of scattered HE
Grade 4a+ Mild-to-moderate deposition of scattered HE
Grade 4b- Moderate deposition of HE with any circinate form
Grade 4b Moderate-to-severe deposition of HE with any circinate form
Grade 4b+ Severe deposition of HE with any circinate form, but fewer than those in standard photograph 4

**Fig. 2:**
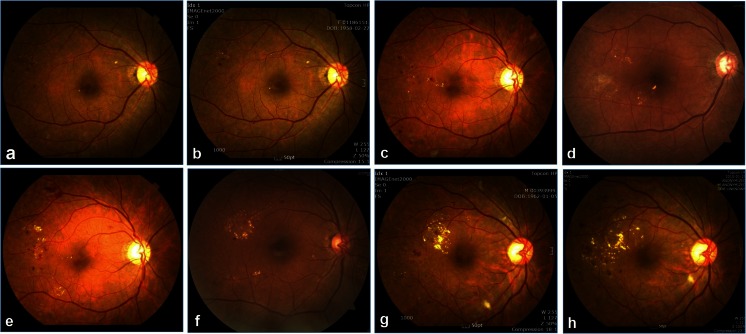
Typical examples of fundus photographs in each grade. **a** Grade 2. **b** Grade 3. **c** Grade 4a-. **d** Grade 4a. **e** Grade 4a+. **f** Grade 4b-. **g** Grade 4b. **h** Grade 4b+

Thus, the fundus photographs yielded a grading of HE that spanned 10 grades overall (1, 2, 3, 4a-, 4a, 4a+, 4b-, 4b, 4b+, 5) (Fig. 2). Fundus photographs were graded in a masked fashion by two independent graders (J. H. S. and S. J. L.) and adjusted by a third grader (H. S. C.) in cases of disagreement.

The secondary efficacy endpoints encompassed improvements in vascular leakage; microaneurysms; hemorrhages; intraretinal microvascular abnormalities at FA, BCVA, IOP and CFT as detected by OCT, and CST.

### Safety endpoints

Vital signs were monitored during each visit while hematology and clinical chemistry were examined at T0 and T12 visits. The laboratory safety tests included tests for the following: red blood cell count (RBC); white blood cell count (WBC) with differential count; platelets; hematocrit (Hct); hemoglobin (Hb); erythrocyte sedimentation rate (ESR); blood creatinine level; aspartate aminotransferase (AST) level; alanine aminotransferase (ALT) level; gamma glutamyltransferase (γGT) level; blood fibrinogen level; activated partial thromboplastin time (aPTT); and lipid panel. We also monitored fasting blood glucose and glycosylated hemoglobin (HbA1c) during each visit. Lastly, possible adverse effects of treatment were monitored by investigators who questioned the patient during each visit and on the final day of assessment.

### Study populations

**Fig. 3:**
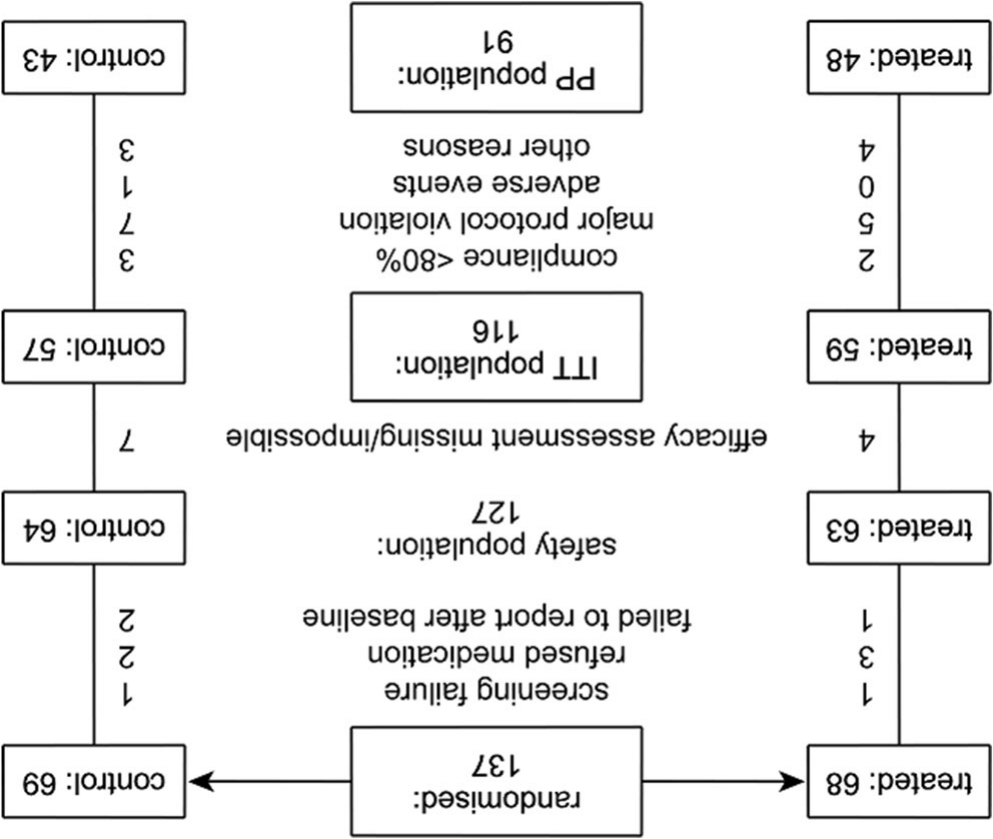
Patients’ dispositions. ITT = intention to treat, PP = per protocol

Figure 3 reports patients’ dispositions. The primary test population was the intention-to-treat (ITT) population, which included all patients who were randomized to treatment, received the medication they had been randomized to, and had both valid baseline and 12-month fundus photography. The secondary test population was the per-protocol (PP) population, which included all patients of the ITT population who, in addition to this, complied with all the inclusion and exclusion criteria and took at least 80 % of the planned treatment doses. The safety population included all the 127 patients who participated in the trial, who received the medication they had been randomized to, and who were seen at least once by the attending physician (Fig. 3).

### Statistical methods

The primary variable was an improvement of HE in NPDR that was defined in this study as a decrease in HE by at least two points on the grading scale. Based on the existing literature, it is estimated that such a decrease could spontaneously occur in approximately 20–30 % of the patients, thus reflecting known fluctuations that exist in the severity of background retinopathy. The study design was consistent with the choice of a two-tailed, chi-square test for significant differences between proportions. An accepted level of significance was *P* ≤ 0.05, with a wanted power of 90 %. Using a sample size of 56 patients per group, the study would have had a power of 90.2 % to yield statistically significant results under the conditions expressed above. In order to account for non-assessable cases and for randomization in blocks, the sample size was increased to 65 patients per group. Chi-square test was accompanied by a relative risk estimate of the proportion of improved patients in the ITT population. To exclude any effect associated with demography, history, and progress of underlying diabetes, the rate of success was also analyzed using binary multivariable logistic regression analysis. The following were included among the putative predictors: baseline HE severity (≤4a/>4a); number of affected eyes; age (<60/≥60 years); sex; body mass index (BMI) (<25/≥25); diabetes duration (<10/≥10 years); as well as the changes - estimated with the last observation carried forward procedure and coded as decreased vs. unchanged or increased - in blood glucose, Hb1Ac, total cholesterol, high density lipoprotein (HDL), low density lipoprotein (LDL), and triglycerides (TG). These analyses were also performed on the PP population.

The secondary efficacy endpoints were only analyzed only in the ITT population.

The safety analysis, performed on the safety group, monitored the following: the course of adverse events, laboratory data, vital signs, and changes in physical examination. All analyses were performed using the Statistical Package for the Social Science version 12.0 for Windows (SPSS Inc., Chicago, IL, USA).

## Results

### Baseline demographic characteristic

There was no evidence of clinical or statistical differences in demographic profile, history of DM, and general or specific medical history, among the treatment groups (Table 2), with the exception of concomitant medications in use (test group, 86.44 %; control group, 70.18 %; *P* = 0.0332). In detail, 91 of 116 patients the in ITT population have administered combining drugs and the major differences between treatment groups were in medications for digestive system and central nervous system.

**Table 2:** Baseline characteristics

Variable		Test (*n* = 59)	Control (*n* = 57)	*P*
Gender	women, n (%)	29 (60.4 %)	19 (39.6 %)	0.084*
Age (yrs)	mean ± std	59.1 ± 8.7	59.9 ± 10.6	0.893†
BMI (kg/m^2^)	mean ± std	23.78 ± 2.98	23.91 ± 2.95	0.827†
Diabetes duration (yrs)	mean ± std	15.4 ± 8.7	14.2 ± 7.5	0.441†
History of other diseases	yes, n (%)	31 (52.5 %)	29 (50.9 %)	0.858*
History of retinopathy	yes, n (%)	57 (96.6 %)	53 (93.0 %)	0.260‡
Previous treatments	yes, n (%)	5 (8.5 %)	1 (1.8 %)	0.207‡
Concurrent treatment	yes, n (%)	51 (86.4 %)	40 (70.2 %)	0.033*

BMI = body mass index, kg = kilogram, m2 = square meter, *n* = number of patients, std = standard deviation, yrs = years

*χ2-test

†Independent *T* test

‡Exact test

### Outcomes

#### Hard exudates

**Fig. 4:**
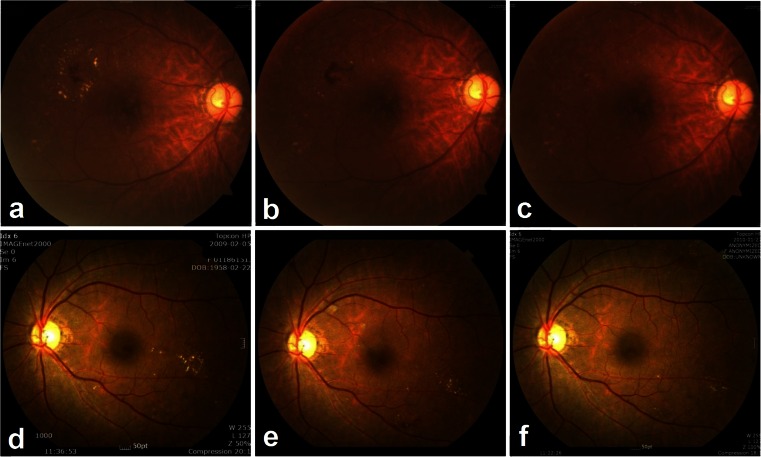
Fundus photographs of two subjects in sulodexide group. **a** At T0, fundus photograph showed grouped hard exudates graded as 4b-. **b** Hard exudates gradually decreased to grade 3 at T6. **c** Further diminution of hard exudates attained grade 2 at T12. **d** Funds photograph of another subject revealed grouped hard exudates of grade 4b- at T0. **e** This patient also showed improvement in hard exudates to grade 4a at T6. **f** Further improvement continued to reach grade 3 at T12

Success, which was defined as an improvement in hard exudates by at least two categories of the severity, was seen in 39.0 % of the affected eyes among the treated patients (95 % confidence interval [CI], 28-50 %) compared to 19.3 % among control patients (95 % CI, 6–33 %). This difference was statistically significant (chi square test, *P* = 0.005; Table 3). This yields a number needed to treat of five (95 % CI, 3–16) to obtain one success more with the tested treatment, in comparison with the standard of care alone. Figure 4 illustrates two paradigmatic cases recorded under treatment (Fig. 4).

**Table 3:** Proportion of success

Population	Control n (%)	Test n (%)	Total n (%)	Chi square
ITT				
Success	16 (19.3 %)	32 (39.0 %)	48 (29.1 %)	
Failure	67 (80.7 %)	50 (61.0 %)	117 (70.9 %)	7.798; *P* = 0.005
Total eyes	83	82	165	
PP				
Success	12 (17.6 %)	28 (38.9 %)	40 (28.6 %)	
Failure	56 (82.4 %)	44 (61.1 %)	100 (71.4 %)	7.732; *P* = 0.005
Total eyes	68	72	140	

ITT = intention-to-treat, *n* = number of eyes, PP = per-protocol

The logistic regression yielded an odds ratio (OR) of 2.790 (95 % CI, 1.155–6.743; *P* = 0.023) for the effect of treatment once adjustments were made for demographic, prognostic and disease confounders, as described in Table 4. The analysis also confirmed some clinically known effects (Table 4). When both eyes were affected, the chance of success was lower (OR 0.430; 95 % CI, 0.190–0.969; *P* = 0.042). Increased cholesterol reduced the chances of success (OR 0.311; 95 % CI, 0.103–0.940; *P* = 0.039), while increased HDL had an opposite effect (OR 2.458; 95 % CI, 1.045–5.780; *P* = 0.039).

**Table 4:** Results of the multivariable logistic regression analysis for the outcome “success”

Variable	ITT population		PP population	
	OR [95 % CI]	*P*	OR [95 % CI]	*P*
Gender (male vs. female)	0.840 [0.379 to 1.863]	0.669	0.915 [0.385 to 2.178]	0.841
Age (≥60 years vs. <60 years)	1.660 [0.742 to 3.712]	0.217	1.787 [0.727 to 4.389]	0.206
BMI (≥25 vs. <25)	1.557 [0.661 to 3.668]	0.311	1.612 [0.597 to 4.357]	0.346
Diabetes duration (≥10 years vs. <10 years)	1.101 [0.415 to 2.926]	0.846	1.372 [0.462 to 4.068]	0.569
Baseline hard exudates severity (>4a vs. ≤4a)	2.134 [0.948 to 4.805]	0.067	1.669 [0.660 to 4.222]	0.280
Affected eyes (both vs. single)	0.430 [0.190 to 0.969]	0.042	0.604 [0.242 to 1.503]	0.278
*Glycaemia (†increased vs. decreased)	0.824 [0.371 to 1.833]	0.636	0.663 [0.264 to 1.664]	0.382
*Hb1Ac (†increased vs. decreased)	0.841 [0.377 to 1.876]	0.672	0.675 [0.259 to 1.757]	0.420
*Cholesterol (†increased vs. decreased)	0.311 [0.103 to 0.940]	0.039	0.279 [0.083 to 0.934]	0.038
*HDL (†increased vs. decreased)	2.458 [1.045 to 5.780]	0.039	3.092 [1.160 to 8.238]	0.024
*LDL (†increased vs. decreased)	2.038 [0.741 to 5.606]	0.168	2.193 [0.727 to 6.617]	0.164
*Triglycerides (†increased vs. decreased)	1.012 [0.428 to 2.394]	0.977	1.532 [0.586 to 4.002]	0.384
Treatment (test vs. control)	2.790 [1.155 to 6.743]	0.023	4.062 [1.432 to 11.523]	0.008

BMI = body mass index, CI = confidence interval, HbA1c = glycosylated hemoglobin, HDL = high density lipoprotein, ITT = intention-to-treat, LDL = low density lipoprotein, OR = odds ratio, PP = per-protocol

* Change in the 12-month observation, estimated with the last observation carried forward procedure

† Increased set includes cases of unchanged values

The PP population yielded almost identical results to the ITT population (Table 3), with a success rate of 38.9 % in treated patients compared to 17.6 % in control patients (*P* = 0.005). In this population the logistic regression analysis (Table 4) confirmed the significant treatment effect (OR 4.062, 95 % CI, 1.432–11.523; *P* = 0.008) as well as the favourable effect of decreased cholesterol and increased/unchanged HDL.

#### Secondary efficacy variables

None of the tested secondary efficacy variables (FA, BCVA, IOP, CFT, and CST) yielded statistically significant differences between the treatment groups.

#### Safety analysis

Overall, 18 out of the 127 patients in the safety population (14.17 %) reported 32 adverse events: seven out of 63 patients in the treatment group (11.11 %) reported 17 reactions; and 11 out of 64 patients in the control group (17.19 %) reported 15 reactions. The proportion of patients with adverse events was not significantly different between treatment groups (chi square, *P* = 0.3263). One treatment patient and two control patients reported potential treatment-related adverse events, all gastrointestinal in nature. One control patient reported a severe event of angina, which was classified as definitely not associated with the assigned treatment.

There were no statistically or clinically relevant evidence of differences between treatment groups with regards to the course of vital signs and laboratory tests.

## Discussion

Diabetic retinopathy is a microvascular complication that primarily affects capillaries, which results in damage to retinal vascular endothelium, leading to leakage and ischemia. Subsequent visual loss is due to the occurrence of hemorrhages, HE, macular edema, and retinal detachment. There are currently several treatment options available for diabetic retinopathy, but no definitely established pharmacological approach has been defined.

Sulodexide is a highly purified GAG with a high affinity for anti-thrombin III, heparin cofactor II, and vascular endothelium [6, 7, 19, 20]. Preliminary observations of patients with diabetic retinopathy that were treated with sulodexide have indicated significant reductions in HE, thus highlighting a potential benefit at the retinal capillary level [14]. Moreover, the incidence and severity of adverse events associated with sulodexide was reported to be no different than those of the placebo group, even at high doses, although mild gastrointestinal upsets were reported [9, 21, 22].

The DRESS research was designed to assess the effectiveness of sulodexide in the treatment of HE in NPDR in type 1 and type 2 DM patients. Vascular complications of DM are generally accompanied by development of endothelial dysfunction or injury, and in experimental models, this endothelial dysfunction or injury is manifested by an increased number of circulating endothelial cells. In vitro and animal models (rats with streptozotocin-induced diabetes) of endothelial injury/dysfunction have reported that sulodexide was able to repair or prevent the endothelial damage, lower the number of circulating endothelial cells, and improve the endothelium-dependent relaxation of small arteries [23–25]. Recently, it was proved that oral sulodexide administration in patients with type 2 DM enhances the availability of precursors for GAG synthesis, thereby improving the endothelial glycocalyx dimension in sublingual and retinal vascular beds. Sulodexide also helped normalization of systemic vascular permeability and GAG metabolism [7].

In this double-masked, randomized, multicenter study, efficacy analysis was performed on a total of 165 eyes in the ITT population and a total of 140 eye in the PP population. The primary endpoint of this study, and defined as an improvement in HE by two categories of severity, was significantly greater among patients receiving sulodexide compared to the control group, both in the ITT and PP populations. In addition to the significant treatment effect, our study displayed a significantly negative effect of high cholesterol and a significantly positive effect of HDL. Other variables reported to be clinically related to changes in the severity of HE, namely blood glucose, HbA1c, LDL, and TG, were not found to be significant predictors of success or failure in this study. This study also showed a favorable safety profile of sulodexide.

The trial recruited diabetes patients with only mild-to-moderate retinopathy and relatively good vision, and hypothesized that sulodexide could be effective at the early phases of exudation, when permanent damage to the vessel wall has not yet occurred. In the presence of permanent organ damage, other metabolic pathways and new pathologic factors such as vascular endothelial growth factor (VEGF) might become activated, which may not respond to the pharmacodynamics of sulodexide. None the less, further investigation of its use in patients with more severe diabetic retinopathy and/or low vision due to macular edema is required. If the results are positive, sulodexide may act as an adjunctive treatment to laser therapy or anti-VEGF agents, which are the current treatment standard.

Although an improvement in HE was significantly greater in the suldoexide group compared to the control group, about 60 % of patients receiving sulodexide did not react to the treatment. It might be partly due to the low dose of study drug and the limited duration of treatment. This study used the lowest daily dose of sulodexide, because of the safety issue. Another explanation for this result might be the limited effect of sulodexide on vessel wall. Sulodexide is postulated to have its beneficial effect on retinal capillary by partial restoration of GAG content. The vascular leakage, resulting in HE, in a diabetic retinopathy is also caused by some other factors, e.g. loosening of endothelial tight junctions induced by VEGF. The use of sulodexide could have decreased the vascular leakage by supplementing GAGs of capillary basement membrane and correcting altered anionic charge. However, there were still other pathogenic factors increasing vascular permeability and not influenced by sulodexide treatment. These might have resulted in partial response to sulodexide.

One of the limitations of this study is the relatively high proportion of ITT patients, 25 out of 116, who did not complete the study according to protocol (Fig. 3). However, we consider it unlikely that this introduced a substantial bias in the evaluation of the drug effect, since the ITT and PP results were comparable, and the proportion of patients and reason for exclusion were very similar between the two treatment groups.

Another limitation is that the dose of sulodexide used in this study has not been validated for the management of diabetic retinopathy. We selected the 50 mg daily dose, a common regimen used in clinical practice that has also been reported in several published clinical trials, mainly for precautionary reasons given the long duration of treatment (1 year). Other studies in diabetes patients used higher doses (100–400 mg per day); however, the duration of treatment in these cases was significantly shorter (4–6 months) [9, 11, 14, 16, 26]. Considering the safety of sulodexide observed in this study, a higher daily dose should be used in any subsequent long-term evaluations of this drug in patients with diabetic retinopathy.

In conclusion, the results of our study indicate that the use of sulodexide, a natural GAG with complex effects on the vascular wall, effected a decrease in macular HE in patients with mild-to-moderate NPDR and the sulodexde treatment group showed significantly greater improvement in HE severity than that shown by the placebo group. However, further and probably larger long-term studies are warranted, possibly with higher dosages, to confirm whether the treatment with sulodexide has a sustained beneficial effect on NPDR.
